# The Wage-Earning Value of the One-Eyed Man

**Published:** 1914-05-23

**Authors:** 


### LEGAL NOTES FOR INSTITUTIONAL WORKERS.

### The Wage-Earning Value of the One Eyed Man.

In the recent case of *Law* v. *Baird and Co.*, 51 S. L. R. 388, the difficult question of the effect of the loss of an eye on a workman's wage-earning capacity was considered; and we propose to state briefly how the law of that matter at present stands. The Workmen's Compensation Act provides that "in the case of partial incapacity the weekly payment shall in no case exceed the difference between the amount of the average weekly earnings of the workman before the accident and the average weekly amount which he is earning *or is able to earn in some suitable employment or business* after the accident." In the case mentioned the applicant was a miner, who when working at the face in one of the defendants' coal-pits was struck by a chip of coal in the eye, as a result of which the eye was injured and eventually he lost the sight of it. The accident admittedly arose out of and in course of the employment, and the employers admitted the man's claim to compensation and paid him compensation for five months, when they ceased payment on the ground that he was able to resume his former occupation as a miner at the face and to earn his former wage. The workman then took action for the continuance of his compensation payments. The Sheriff (County Court Judge) found in favour of the employers and dismissed the man's application. An appeal was taken to the Inner House of the Court of Session, who have affirmed the judgment of the inferior Court.

### The Right to Choose One's Work.

The appeal raised several purely legal questions, such as the personal choice of the workman, his right to exercise his own judgment as to the suitability of the work offered him, and so on, but here we shall only refer to points of interest to the hospital man as well as to the lawyer. In the first place, the decision recognised the principle that it is a question of fact whether a workman is able to take suitable employment, and the other points are really subsidiary to that principle. They are: (*a*) whether the risk to a one-eyed man formerly employed—when he had two eyes—as a miner was such as to entitle him to refuse to resume work as a miner; and (*b*) whether the fact that a similar accident might involve total loss of eyesight ought not to be considered in determining the question of suitability of employment.

As to the first point, the Court came to the firm conclusion on the evidence, in which presumably medical evidence was the chief factor, that while a chip flying from the pick might strike the other eye and seriously injure it, in point of fact a miner was often struck by a flying chip without any injury resulting; that was an ordinary risk incident to this workman's employment as a miner and was not increased by the fact that he had lost the sight of one of his eyes. But, even so, then the argument for the workman was that if his other eye was injured by a flying chip the consequences would be so very serious for him—total loss of sight—that that contingency made employment at the face always an unsuitable employment for a one-eyed miner. That consideration, however, the Court ruled as irrelevant, for, they pointed out, to take it into account would be to make the man's present employers continue to pay him compensation in respect of a contingency which might never take place. And, it was further laid down, if an accident did in future occur to the other eye causing total blindness, the workman still had a remedy. He could recover compensation for total incapacity in respect of such second accident.

### Effect of the Verdict.

The result practically of this case is that if it is proved to the Court that a workman who has lost an eye is able to resume his former occupation at his former wage without increase of risk to himself his compensation will be stopped. This seems rather hard on the employee, for suppose on resuming work he is after a time dismissed or suspended through dull trade, his one-eyed condition then is a great handicap to him.

